# The gut microbiota and microbial metabolites are associated with tail biting in pigs

**DOI:** 10.1038/s41598-021-99741-8

**Published:** 2021-10-15

**Authors:** Else Verbeek, Linda Keeling, Rikard Landberg, Jan Erik Lindberg, Johan Dicksved

**Affiliations:** 1 Department of Animal Environment and Health, Swedish University of Agricultural Sciences, Box 7068, 750 07 Uppsala, Sweden; 2 Department of Biology and Biological Engineering, Food and Nutrition Science, Chalmers University of Technology, Gothenburg, Sweden; 3 Department of Animal Nutrition and Management, Swedish University of Agricultural Sciences, Box 7024, 750 07 Uppsala, Sweden

**Keywords:** Animal behaviour, Animal physiology, Microbiology, Zoology

## Abstract

Tail biting is an abnormal behaviour that causes stress, injury and pain. Given the critical role of the gut-microbiota in the development of behavioural problems in humans and animals, the aim of this study was to determine whether pigs that are biters, victims of tail biting or controls (nine matched sets of pigs) have a different microbiota composition, diversity and microbial metabolite profile. We collected faecal and blood samples from each individual for analysis. The gut microbiota composition was most different between the biter and the control pigs, with a higher relative abundance of Firmicutes in tail biter pigs than the controls. Furthermore, we detected differences in faecal and plasma short chain fatty acids (SCFA) profiles between the biter and victim pigs, suggesting physiological differences even though they are kept in the same pen. Thus, in addition to supporting an association between the gut microbiota and tail biting in pigs, this study also provides the first evidence of an association between tail biting and SCFA. Therefore, further research is needed to confirm these associations, to determine causality and to study how the SCFA profiles of an individual play a role in the development of tail biting behaviour.

## Introduction

Despite decades of research, tail biting is still a major problem in intensive pig production. Tail biting has serious consequences for the animals involved because it leads to physical injury and pain as well as psychological stress^1,2^. Tail biting also has a negative impact on the economic performance of pig farms^3^. The EU Council directive 2008/120/EG bans routine tail docking, but allows exemptions to prevent individual animal damage. However, a total ban on tail docking currently exists in only three European countries (Norway, Sweden and Finland) and it is estimated that 80–99% of pigs are tail docked in other European countries^3^.

There is no single cause of tail biting, and there is an extensive amount of literature identifying different causes and risk factors as well as numerous investigations of different solutions (reviewed in^4^). A generally accepted theme is that tail biting is an abnormal behaviour, that is expressed under barren conditions, and results from the inability to fulfil the natural need for rooting, foraging and explorative behaviours^5,6^. In addition, domestic pigs are exposed to many stressful challenges throughout their lives, and several of these have been identified as risk factors for the development of tail biting. These factors can be environmental, such as temperature^7^, feeding method^7^, lack of appropriate enrichment^8^, and inappropriate pen design e.g., fully slatted floors^7,9^. Also several social factors such as insufficient space at the feeder leading to social competition^9,10^ and high stocking density^9^ are risk factors for tail biting, as are individual factors such as nutritional status^11^ and neurobiology^12^. However, it is currently not fully understood how all these different factors contribute to the development of tail biting.

One factor that could provide the link between food-related behaviours, different types of stressors and tail biting, is the gut microbiota^11^. The gut microbiota plays a major role in regulating homeostatic processes in the host, such as the immune system, the cardiovascular system, the digestive system and metabolic processes^13^. The gut microbiota also influences key brain processes, and this link between the gut and the brain is called the microbiota-gut-brain axis^14^. Rodents raised without a gut microbiota (germ-free) showed increased physiological stress responses to restraint^15^, and it is now recognized that the gut microbiota is a critical component in the regulation of the Hypothalamus–Pituitary–Adrenal (HPA)-axis^16^. Germ-free mice also showed socially impaired and repetitive behaviours^17^, suggesting that the gut microbiota is important for the development of normal social behaviour. On the other hand, stressful experiences (including social stress) alter gut microbiota structure^18^ and reduce diversity^19^. Providing faecal matter from healthy animals or supplementing with probiotics can reverse some of the abnormal social behaviours in animals with an unbalanced gut microbiota^20,21^. In humans, it is now thought that an unbalanced gut microbiota contributes to the development of a range of abnormal behaviours and can be a contributing factor to depression and anxiety disorders^22–24^.

Intensively reared pigs are kept under tightly controlled environmental conditions, fed a standardized diet to promote fast growth, and may be given antibiotics. They are also kept indoors under strict hygienic conditions, which compromises the normal development of the gut microbiota due to a lack of exposure to environmental microbes^25,26^. Furthermore, the multiple stressors experienced already from an early age (e.g., weaning and separation from the dam, castration, frequent mixing with unfamiliar animals) may pose an increased risk of developing an unbalanced gut microbiota. A first explorative study has shown that pigs involved in tail biting had a different microbiota composition compared to pigs not involved in tail biting, and this difference was primarily dependent on a reduced abundance of *Lactobacillus* in the animals involved in tail biting^27^.

One of the pathways for crosstalk between the gut and the brain is through the production of microbial metabolites that have neuroactive properties. Short-chain fatty acids (SCFA) are the main metabolites produced in the large intestine by anaerobic bacterial fermentation of dietary fibre^28^. Acetate, propionate, and butyrate are the main SCFA in the colon and are key components in gut-brain communication through a number of signalling pathways, including the stimulation of neuropeptides from enteroendocrine cells (e.g., glucagon-like peptide 1 (GLP-1), peptide YY, serotonin, and GABA) through which they can influence behaviour^28^. A reduction in SCFA producing bacteria has been identified as a key factor in dysbiosis of the gut microbiota and is linked to neuropsychiatric disorders in both humans and in animal models^29^. In humans^30^ and monkeys^31^, faecal SCFA concentrations were lower in individuals with depressive symptoms compared to healthy controls. SCFA concentrations were also lower in children diagnosed with autism spectrum disorder^32^, which is characterized by repetitive behaviours and impaired social interactions. Even though faecal SCFA are usually measured as a proxy of colon-derived SCFA, only approximately 5% is excreted in the faeces while 95% of colonic SCFA is readily absorbed in the colon in mammals^28,33,34^. SCFA from the periphery can also cross the blood–brain-barrier^28^. How the gut-microbiota and SCFA influence behaviour in pigs has so far received little attention. The novelty of this study is that we will investigate for the first time whether there is an association between faecal and plasma SCFA profiles and abnormal behaviour in pigs.

Given the critical role of the gut-microbiota in the development of behavioural problems in humans and animals, the aim of this study was to determine whether matched sets of pigs that are biters, victims of tail biting or controls, i.e., pigs not involved in a tail biting episode, have a different microbiota composition, diversity and SCFA profile. We expect differences between the biter and victim pigs compared to the control pigs, given the stress of being involved in tail biting, but differences between biters and victims might support the hypothesis that an unbalanced gut-microbiota and an altered SCFA profile plays a role in the development of tail biting.

## Methods

### Animal ethics

All methods were carried out in accordance with relevant guidelines and regulations. The study was approved by Uppsala animal ethics committee (document numbers C105416/16) and complied with the ARRIVE guidelines^35^.

### Animals and housing

In total, 29 pigs were included in the study, of which ten were biters, nine were victims and ten were controls. The pigs were selected to belong to nine matched sets, each set consisting of at least one biter, one victim and one control. Matching appropriate controls to the biter and victim pigs increases the efficiency of the study, and therefore increases the likelihood of finding consistent differences even with a relatively low number of tail biting episodes^36^. Our original aim was to include only pigs from an experimental farm, but after one year we had only identified a few episodes of tail biting. Therefore, we also included pigs from a commercial farm to increase the number of animals available. In total, seven episodes of tail biting on the experimental farm (23 animals) and two episodes of tail biting (six animals) on the commercial farm were identified over a 2-year period.

The experimental farm housed specific pathogen free crossbreed sows of Swedish Landrace, Yorkshire and Hampshire. All pigs had intact tails and were housed in groups no larger than ten finishing pigs, in pens with partially slatted floors (3.6 × 2.2 m). A small amount of straw was given as enrichment but no bedding was provided. The pigs were fed a standard commercial diet (Lantmännen Delta F4106) for finishing pigs, see also Table 1.

**Table 1:** Diets on the experimental and commercial farms.

Component	Experimental farm	Commercial farm
NE (MJ/kg DM)	10.6	11.7
Crude protein (CP, g/kg DM)	160	158
Lysine (g/kg DM)	9.2	10.2
Threonine (g/kg DM)	6.1	6.7
Methionine (g/kg DM)	2.7	2.8
Ca (g/kg DM)	8.0	7.4
P (g/kg DM)	4.7	3.5

The pigs on the commercial farm were crossbreeds between Swedish Landrace, Yorkshire and Hampshire. All pigs had intact tails and were housed in groups of 10–11 pigs in pens with partially slatted floors (3.4 × 2.3 m). A small amount of straw was given as enrichment but no bedding was provided. The diet is indicated in Table 1.

### Selection of pigs

The farm staff identified pens with tail biting problems by monitoring pigs for visible signs of tail injuries on a daily basis during routine cleaning of the pens. In case there was visible tail damage, the farm staff were instructed to conduct direct observations at the home pen for 15 min to identify the biter. For ethical reasons, all biter pigs were removed from the pen as soon as possible to prevent further damage and suffering. Tail biting was defined as one pig having the tail of a conspecific in its mouth resulting in a physical reaction (squealing, grunting, moving away) from the conspecific. A pig was considered a biter when it was clearly observed biting the tail of a pig with visible tail damage at least five times within the 15 min observation time, similar to previous studies^37–39^. In case the farm staff could not clearly identify the biter, the researchers would observe the pigs for 20 min sessions until a biter was identified (minimum of five bites to the tail of a pig with tail injuries). A victims was identified by the presence of tail injuries, and was selected from the same pen as the biter pig. The severity of the tail biting was scored for each victim according to the scoring system developed by Sutherland^40^, based on tail length (scored from 1 to 5), presence of injuries (scored from 1 to 4) and presence of blood (scored from 1 to 4). We then summed the scores for each individual pig, and victims with tail damage scores between 0 and 5 points were considered mild, 5–10 points moderate and more than 10 points severe. Because tail biting in pigs is often mutual, with 80–98% of pigs in the pen involved in tail biting^37^, we chose to select the control pigs randomly from a nearby pen with pigs of similar ages and the same sex as the biters and victims (matched controls) but without any animals with tail damage in the control pen. The pens from which the control pigs were selected had no pigs with tail injuries but were in the same room of the barn and therefore had the same management, diet and environment as the pen with the biter and victim pigs.

### Sample collection and analysis

Faecal and blood samples (EDTA vacutainer) were collected between one (experimental farm) and five days (commercial farm) after victims and biters had been identified. Samples were placed on ice directly after collection and transported to the lab where blood was centrifuged (2000*g* for 10 min at 4 °C) and plasma and faecal matter stored at − 80 °C until analysis. The sample analysis was done by different experimenters than the ones collecting the samples, and the experimenters analysing the samples were blind to the treatments.

### Analysis of the microbiota

DNA was extracted from faecal samples using QIAamp DNA Stool Minikit (Qiagen, Hilden, Germany). The DNA isolation followed the instructions of the manufacturer, but with an extra mechanical lysis step, using 0.1 mm Zirconium/Silica beads (Biospec products, Bartlesville, USA), 2 × 1 min at 6000 rpm with a Precellys evolution (Bertin Instruments, Montigny-le-Bretonneux, France). The isolated DNA was stored at − 20 °C until analysis. 16S rRNA gene amplicons were generated from the V3 and V4 region using the primers (341F 5′-CCTACGGGAGGCAGCAG-3′ and 806R 5′-GGACTACNNGGGTATCTAAT-3′). Sequencing libraries were generated using NEB Next^®^ Ultra™ DNA Library Prep Kit for Illumina (NEB, USA) and the amplicon library was sequenced on an Illumina HiSeq 2500 platform at Novogene. The generated paired-end reads were merged with FLASH (V1.2.7, http://ccb.jhu.edu/software/FLASH/)^41^ and assigned to each sample according to the sample specific barcodes. Quality filtering of sequence data was performed with QIIME (V1.7.0)^42^ and UCHIME^43^ was used to detect and remove chimeric sequences^44^. The reads were clustered using Uparse software (Uparse v7.0.1001)^45^ and OTUs (Operational Taxonomic Units) were generated based on 97% sequence homology.

### Analysis of short chain fatty acids

Faecal SCFA were analysed in 0.5 g faecal matter diluted in 1 mL water, as previously described^46^, using an HPLC system consisted of an Alliance 2795 separations module and 2414 RI Detector (Waters Corp. Milford, MA, USA). Column packet ReproGel H 9µ 300*8 mm was used as the separation column and a ReproGel H, 9µ 30*8 mm (Dr. A. Maisch, Ammerbuch, Germany) was used as a pre-column. SCFAs were analyzed in plasma by LC–MS according to a method described earlier, but with some minor modifications. For detailed description of the analytical procedure for the SCFA analysis, see Supplementary File 1.

### Statistical analysis

The microbial alpha diversity within samples was assessed on OTU level data. Principal coordinate analysis (PCoA) with Bray Curtis distance metrics was used to assess the relationship in microbiota composition between samples. In addition, cluster analyses were done for the individual sets and were based on Bray Curtis distances and UPGMA algorithms. The multivariate analyses were executed on OTU level data using the software PAST (version 4.02)^47^.

The relative abundance of the different phyla, orders, genera and families, and alpha diversity index data were analysed by a non-parametric Friedman test fitting category of pig as a fixed effect and with set as a blocking factor in r (version 4.0.2.)^48^. Because there were two sets with four animals, one animal in the duplicated category was randomly excluded to form nine sets of three animals each (victim, control and biter). To reduce the number of tests, only taxa present in 50% of the samples and with an average abundance of more than 0.1% were included. Both unadjusted and Benjamini–Hochberg False Discovery Rate (FDR) adjusted p-values are presented. Posthoc testing was done when the unadjusted p-value was less than 0.05 using an asymptotic general symmetry test (coin^49^ and multcomp packages^50^), and p-values were Bonferroni corrected.

**Figure 1:**
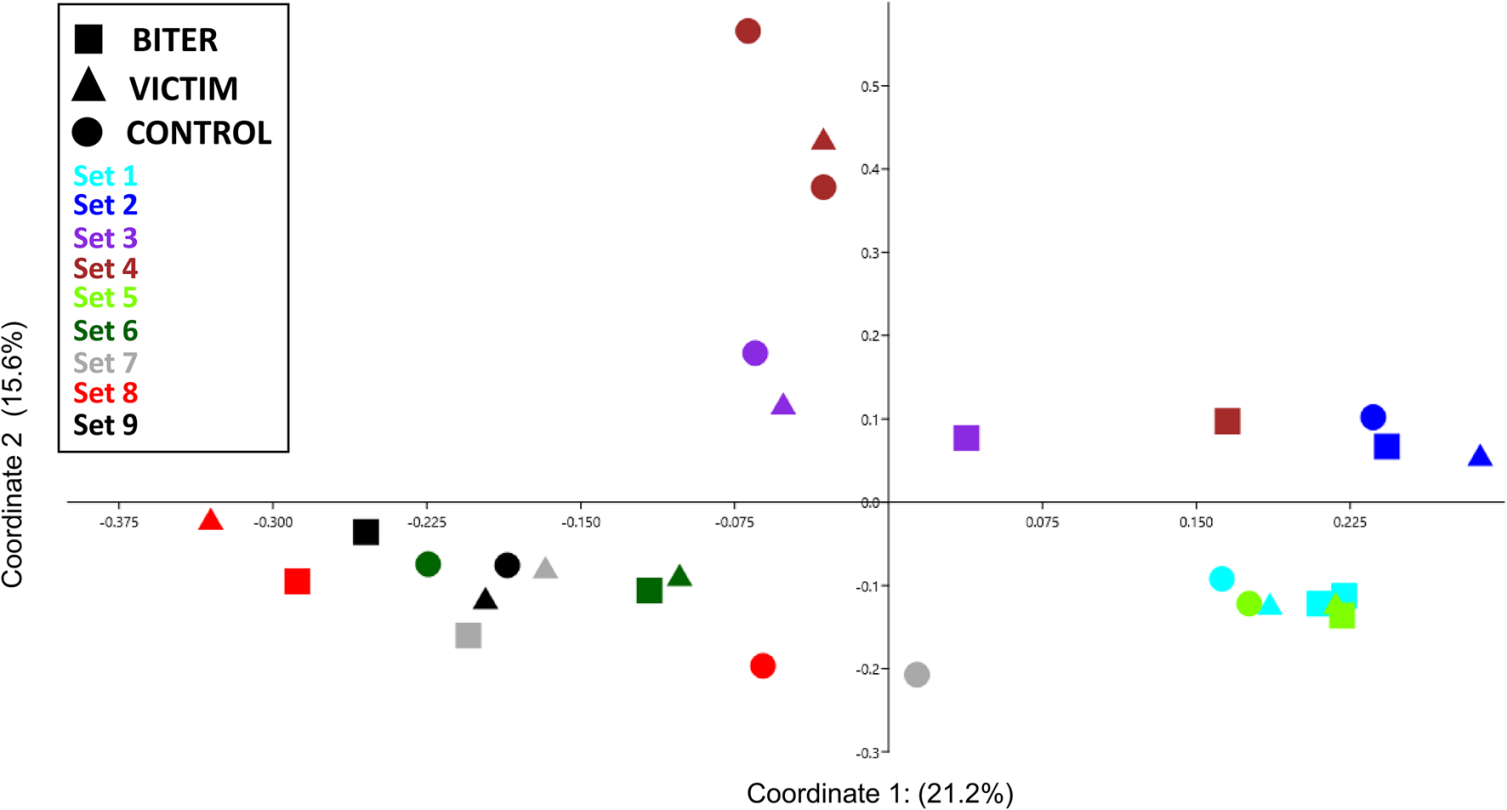
A principal coordinate analysis plot based on Bray Curtis distances showing the associations between samples from their microbiota profiles in faecal samples. Different symbols represent the category of the animals and different sets are indicated by different colours. Sets 2 and 3 represent samples from the commercial farm whereas the remaining samples are derived from the experimental farm. The percent of the data explained by each axis is also shown.

**Figure 2:**
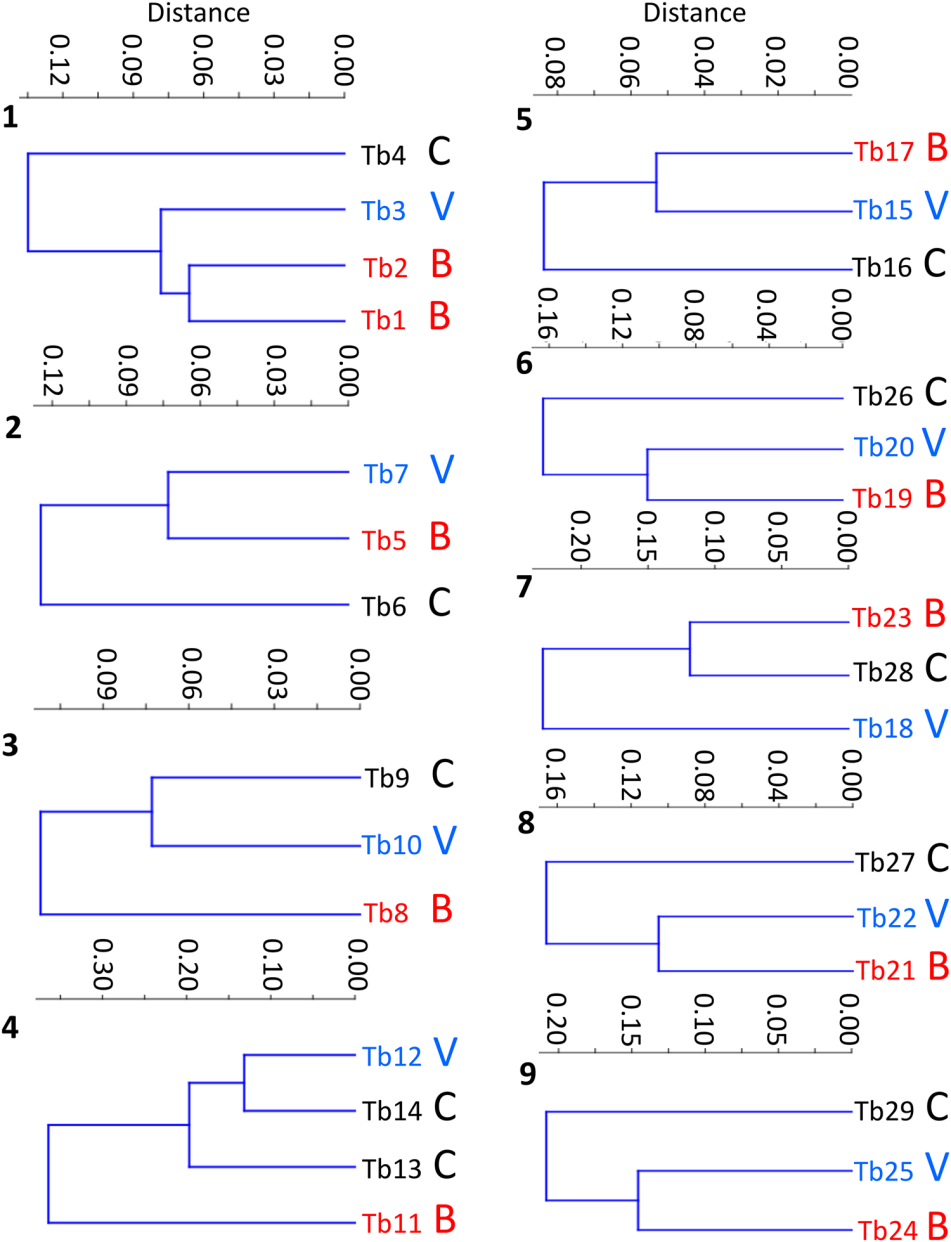
Cluster analysis from the individual matched sets (1–9). The cluster analysis was based on Bray Curtis distances and different colours represent the different categories of pig; biter (B), control (C) and victim (V).

**Figure 3:**
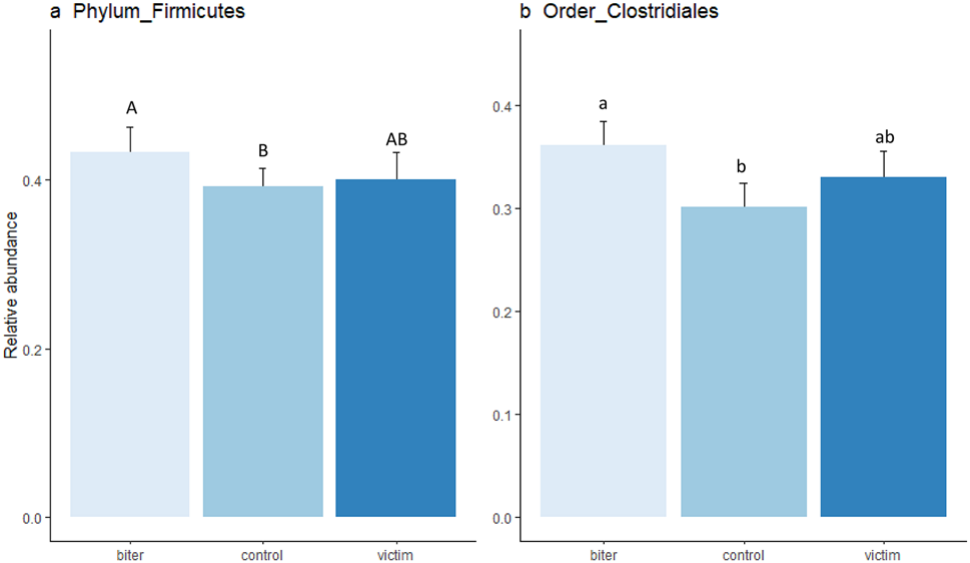
Relative abundance (mean ± sem) of different (**a**) phyla and (**b**) order that were statistically significant for category of pig. Bars with different subscripts indicate Bonferroni corrected statistical significance from the post hoc tests between categories of pigs, with upper case letters indicating significant differences at p < 0.01 and lower case at p < 0.05.

**Figure 4:**
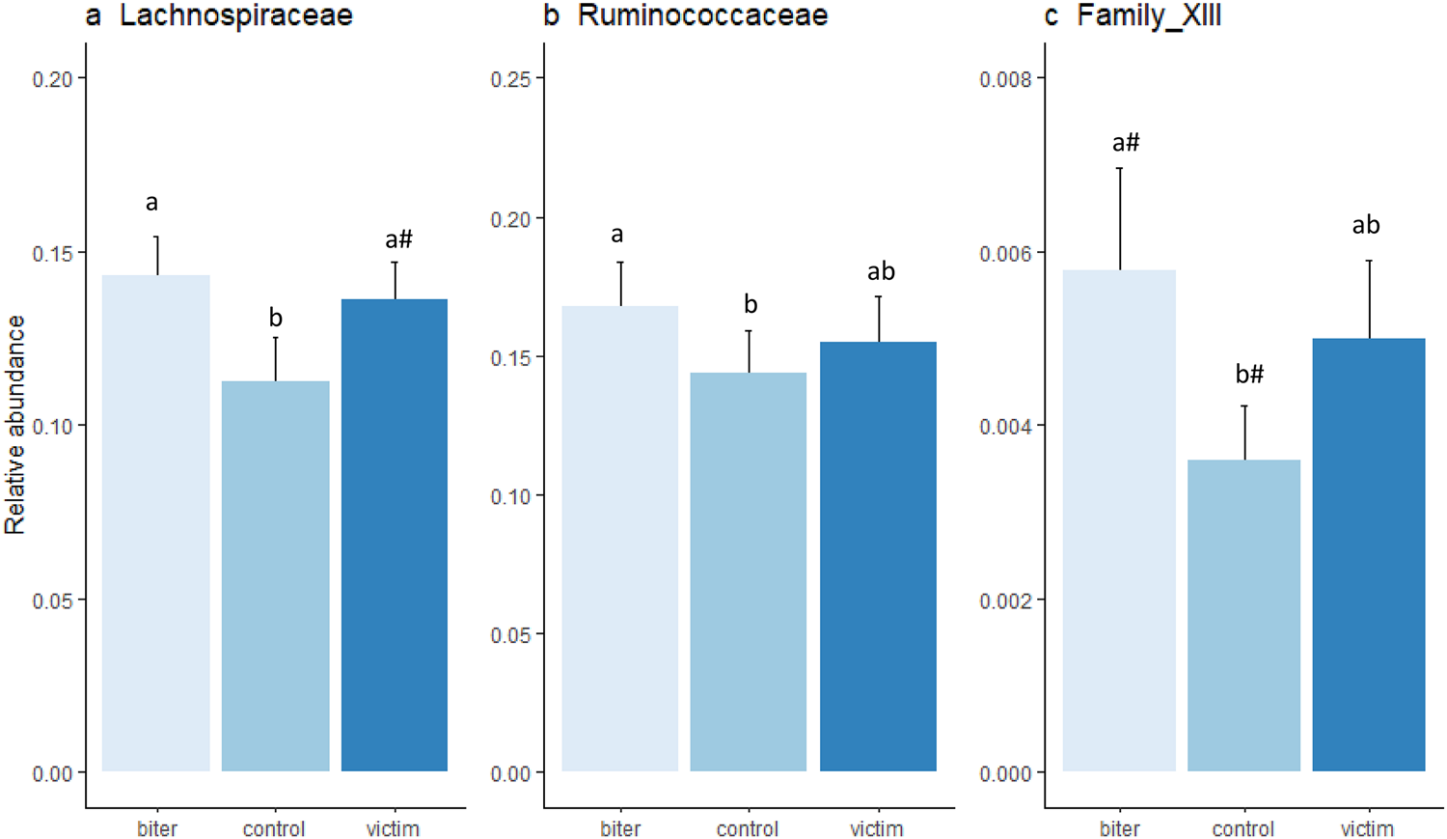
Relative abundance of different families (mean ± sem) that were statistically significant for category of pig. (**a**) Lachnospiraceae, bars with different subscripts indicate Bonferroni corrected statistical significance from the posthoc tests between categories (p < 0.05) and ^a#^a tendency between victim and control (p < 0.1); (**b**) Ruminococaceae, bars with different subscripts indicate a difference between categories (p < 0.05); (**c**) Family_XII, ^#^a tendency between biter and control (p < 0.1).

**Figure 5:**
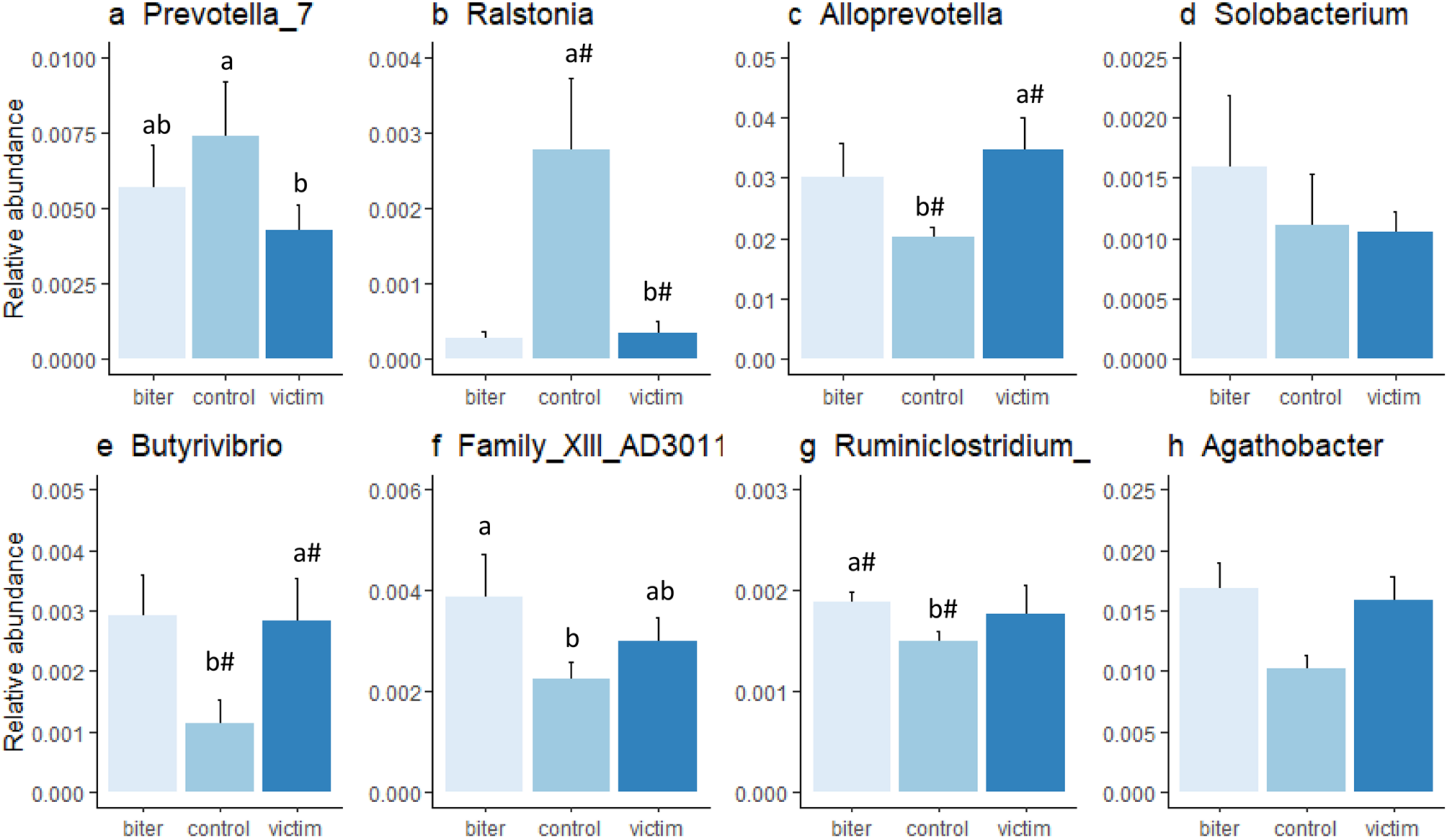
Relative abundance of different genera (mean ± sem) that were statistically significant for category of pig, with (**a**) *Prevotella_7*, bars with different subscripts indicate Bonferroni corrected statistical significance from the posthoc tests between categories (p < 0.05); (**b**) *Ralstonia*, ^#^a tendency for a difference between victim and control (p < 0.1); (**c**) *Alloprevotella*, ^#^a tendency for a difference between victim and control (p < 0.1); (**d**) *Solobacterium*; (**e**) *Butyrivibrio*, ^#^a tendency for a difference between victim and control (p < 0.1), (**f**) *Family_XIII_AD3011*, bars with different subscript indicate a significant difference (p < 0.05); (**g**) *Ruminiclostridium*, ^#^a tendency for a difference between biter and control (p < 0.1); (**h**) *Agathobacter*.

**Figure 6:**
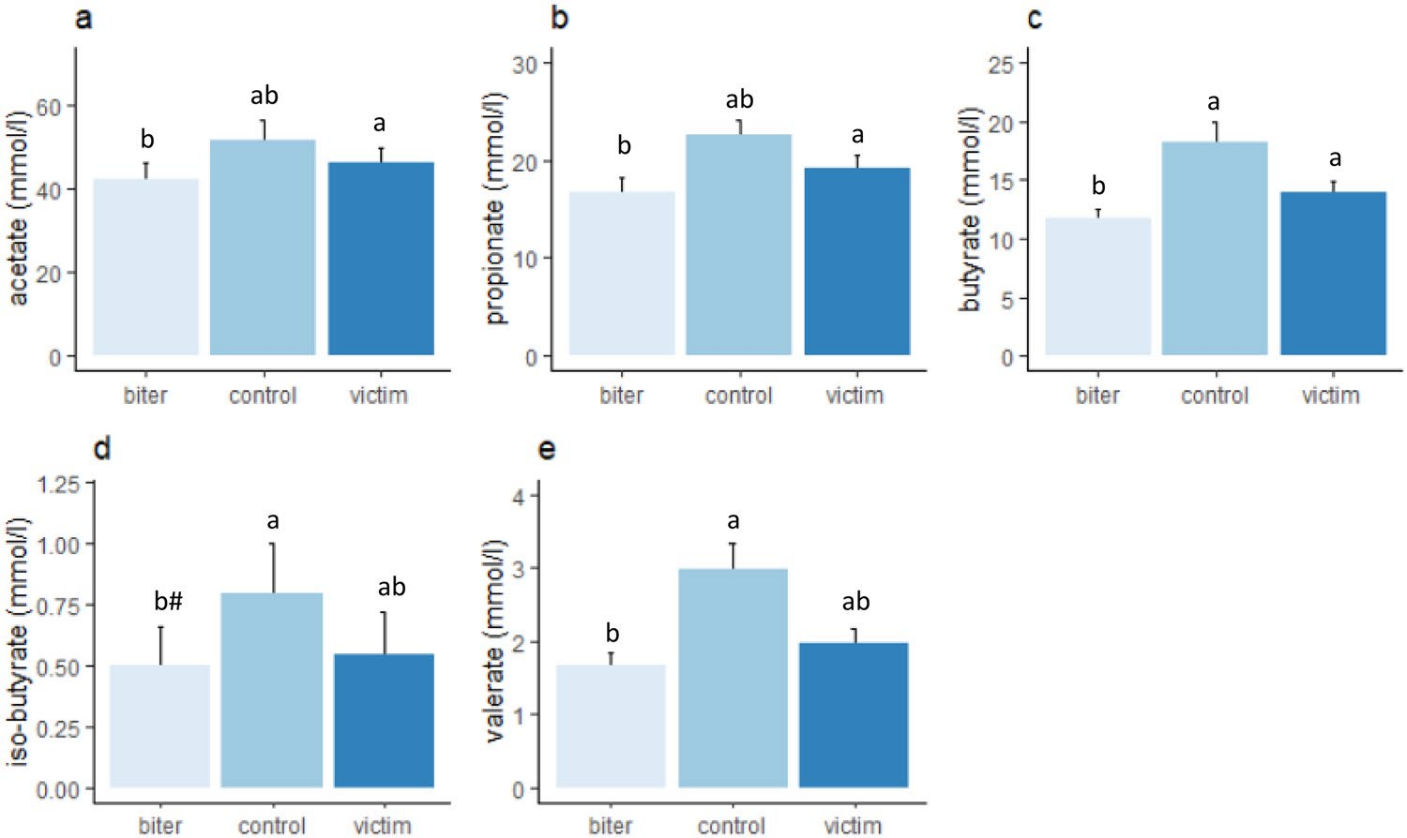
Faecal SCFA (mean ± sem) in the biters, victims and control pigs, with (**a**) acetate, (**b**) propionate, (**c**) butyrate, (**d**) iso-butyrate, (**e**) valerate. Lower case letters indicate significant differences between category of pig, ^#^a tendency for a difference (p < 0.1).

**Figure 7:**
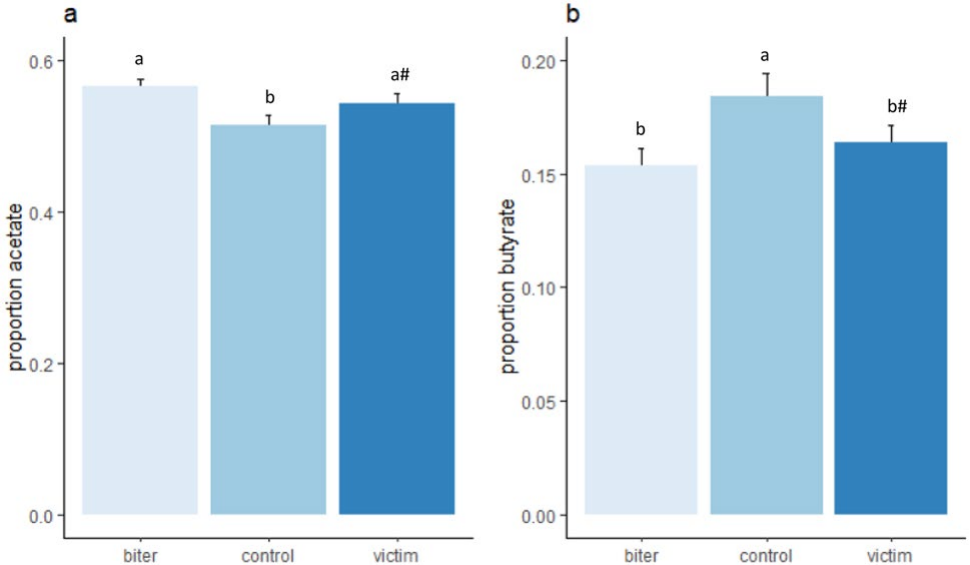
Molar proportions of faecal SCFA (mean ± sem) in the biters, victims and control pigs, with (**a**) the molar proportion of acetate and (**b**) the molar proportion of propionate. Lower case letters indicate significant differences between category of pig. ^#^A tendency of a difference (p < 0.1) between category of pig.

**Figure 8:**
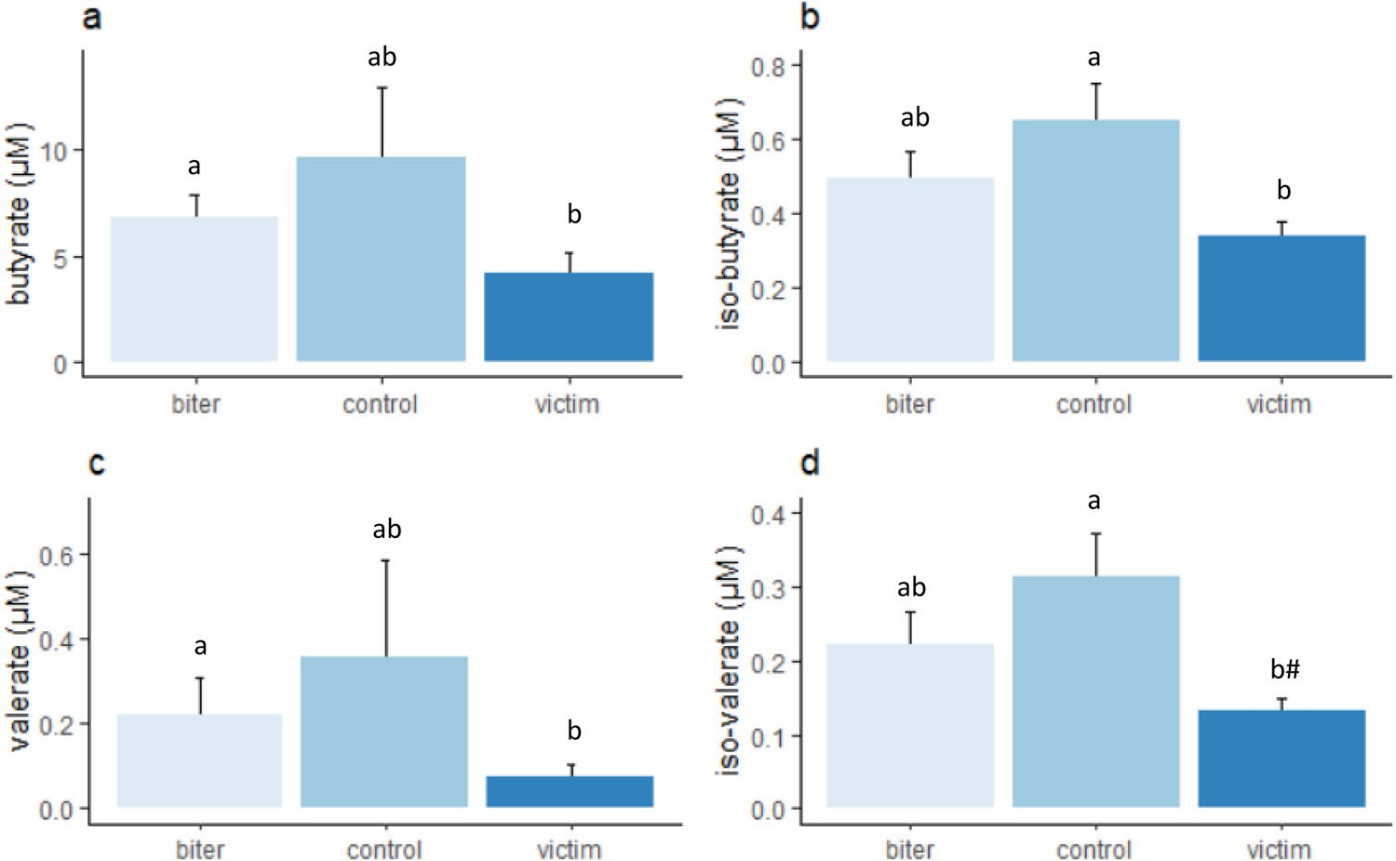
Plasma SCFA (mean ± sem) in the biters, victims and control pigs, with (**a**) Butyrate, (**b**) iso-butyrate, (**c**) valerate, and (**d**) iso-valerate. Lower case letters indicate significant differences between category of pig, ^#^a tendency of a difference (p < 0.1) between category of pig.

SCFA data were analysed in r (version 4.0.2.) and model assumptions of normality and homoscedasticity of the data were visually checked by QQ-plots (LMERConvenienceFunctions package^51^). SCFA were analysed by mixed models (packages lme4^52^ and lmerTest^53^), with category of pig, farm, sex and age as fixed effects and the specific matched set that the animals belonged to and pen as random effects. If the data did not meet the normality assumptions, a data transformation was applied first (log-transformed variables: faecal acetate, iso-butyrate, valerate; faecal proportions of iso-butyrate; all plasma SCFA; and plasma proportions of acetate, butyrate, propionate, iso-valerate). Faecal acetate concentrations still did not meet normality assumptions, and one outlier with a residual larger than 2.5 units was excluded from the analysis. After data transformation and exclusion of the outlier, all variables met the normality assumptions. Non-significant terms were dropped from the model in the final analysis. Posthoc Tukey HSD tests were performed using the emmeans package^54^. The data presented in Figs. 1 and 2 were plotted in the software PAST, and Figs. 3, 4, 5, 6, 7 and 8 were plotted using the package ggplot2 in r^55^.

## Results

### Animal selection

In total, we included nine sets of pigs matched for sex and age, with seven sets of three animals and two sets of four animals (Table 2). In set nine (pen 213, Table 2), we could not clearly identify the biter, even after multiple observation sessions. One pig in pen 213 was biting body parts other than the tail. Pigs that tail bite also perform more abnormal behaviours that involve biting other body parts^38,39^. No other pig in pen 213 was observed to perform any biting behaviour. Therefore, it is very likely that the suspected biter pig in set 9 was the actual tail biter pig.

**Table 2:** Matched sets of pigs engaged in tail biting and controls.

Set	Pig ID	Sex	Age (days)	Farm	Category	Pen #	Tail damage
1	TB1	Female	104	Experimental	Biter (victim)	105	Mild
1	TB2	Female	104	Experimental	Biter	105	
1	TB3	Female	104	Experimental	victim	105	Moderate
1	TB4	Female	103	Experimental	Control	106	
2	TB5	Male	164	Commercial	Biter	403	
2	TB6	Male	164	Commercial	Control	443	
2	TB7	Male	164	Commercial	Victim	403	Moderate
3	TB8	Male	95	Commercial	Biter	222	
3	TB9	Male	95	Commercial	Control	221	
3	TB10	Male	95	Commercial	victim	222	Moderate
4	TB11	Female	119	Experimental	Biter	105	
4	TB12	Male	119	Experimental	Victim	105	Severe
4	TB13	Female	119	Experimental	Control	106	
4	TB14	Male	119	Experimental	Control	106	
5	TB15	Male	109	Experimental	Victim	706	Moderate
5	TB16	Male	109	Experimental	Control	701	
5	TB17	Male	109	Experimental	Biter	706	
6	TB19	Male	70	Experimental	Biter (victim)	312	Moderate
6	TB20	Male	70	Experimental	Victim	312	Severe
6	TB26	Male	70	Experimental	Control	311	
7	TB18	Male	70	Experimental	Victim	312	Severe
7	TB23	Male	70	Experimental	Biter	312	
7	TB28	Male	70	Experimental	Control	310	
8	TB21	Male	70	Experimental	Biter (victim)	312	Severe
8	TB22	Male	70	Experimental	Victim	312	Severe
8	TB27	Male	70	Experimental	Control	311	
9	TB24	Male	70	Experimental	Biter^a^	313	
9	TB25	Male	69	Experimental	Victim	313	Severe
9	TB29	Male	70	Experimental	Control	310	

^a^The biter in set 9 could not be identified with certainty according to our definition of a tail biter pig. Three individuals identified as a biter had wounds on their tail and so were also classified as victims.

### Composition and diversity of the microbiota

The sequence analysis generated on average 83,510 (range 32,981–119,580) sequences/sample. The sequence data was dominated by Firmicutes (average relative abundance; 40.9%), Bacteroidetes (39.3%) and Proteobacteria (12.3%). The alpha-diversity using Shannon (*p* < 0.05) and Simpson (p < 0.05) diversity indexes were associated with tail biting category (Table 3).

**Table 3:** Alpha-diversity indexes (mean ± sem) of the faecal microbiota for biter, victim and control pigs and corresponding statistics.

Index	Biter	Control	Victim	Friedman *χ*^*2*^	p value
Shannon	7.7 ± 0.2^a#^	7.3 ± 0.1^b#^	7.9 ± 0.3^ab#^	8.2	p< 0.05
Simpson	0.98 ± 0.00^a^	0.95 ± 0.02^b^	0.97 ± 0.01^ab^	8.4	p< 0.05
Chao1	1433 ± 53	1530 ± 94	1381 ± 73	0.67	ns
PD whole tree	88.0 ± 2.5	95.9 ± 3.9	85.1 ± 2.9	0.89	ns

^a^Columns with different superscript are significantly different (p < 0.05).

^a#^Columns with different superscripts tend to be significantly different (p < 0.1).

The assessment of the Beta diversity using PCoA revealed a primary clustering of samples that was dependent on the set, although with some overlap between the sets within animals located on the same farm (Fig. 1). The samples from the commercial farm (set 2 and 3 in Fig. 1) clustered together with the samples collected from the experimental farm. The PCoA also revealed that the biters and controls were generally the samples with the largest distance within the sets. A cluster analysis was therefore performed for each set separately. When comparing the pattern of biters, victims and controls within each set, there was a larger distance between biters and controls compared with biters and victim for six of the nine matched sets, so confirming a different microbiota composition in animals involved in tail biting (Fig. 2).

### Relative abundance of different phyla, orders, genera and families

Univariate statistical tests were applied in order to identify specific taxa that differed in relative abundance between animals involved in tail biting and controls. The relative abundance of the phylum Firmicutes was the only variable that was significant after applying the FDR adjustment, (Friedman χ^2^ = 11.55, adjusted p < 0.05 and unadjusted p < 0.01, Fig. 3a), with a higher relative abundance of Firmicutes in the biters than the controls (Bonferroni corrected posthoc test p < 0.01) while victims were not different from controls or biters. None of the other variables reached statistical significance after FDR adjustment, so we present only the non-adjusted p-values here. At the order level, the relative abundance of Clostridiales, which is a member of the Firmicutes phylum, was associated with tail biting status (Friedman χ^2^ = 9.55, p < 0.01, Fig. 3b), with a higher relative abundance in the biters than the controls (p < 0.05).

At the family level, three different families belonging to the Clostridiales order were associated with tail biting. The first was Lachnospiraceae (Friedman χ^2^ = 10.89, p < 0.01, Fig. 4a), with a higher relative abundance in the biter (p < 0.05), and a tendency for a greater abundance in the victims (p = 0.08) than the controls. Ruminococcaceae (Friedman χ^2^ = 9.56, p < 0.01, Fig. 4b) also had a greater abundance in the biters than the controls (p < 0.01). Clostridiales Family_XIII (Friedman χ^2^ = 6.89, p < 0.05, Fig. 4c) also had a greater abundance in the biters than controls (p < 0.1).

At the genus level, *Prevotella*_7 relative abundance was associated with category of pig (Friedman χ^2^ = 12.23, p < 0.01, Fig. 5a), with a lower relative abundance in the victims than the controls (p < 0.01). *Ralstonia* abundance (Friedman χ^2^ = 8.22, p < 0.05, Fig. 5b) tended to be lower in the victims compared to the controls (p = 0.08). *Alloprevotella* relative abundance (Friedman χ^2^ = 6.89, p < 0.05, Fig. 5c) tended to be higher in the victims than controls (p = 0.08). *Solobacterium* (Friedman χ^2^ = 6.22, p < 0.05, Fig. 5d) and *Agathobacter* (Friedman χ^2^ = 6.22, p < 0.05, Fig. 5h) were associated with category of pig, but no contrasts were significant after Bonferroni correction. The relative abundance of *Butyrivibrio* was associated with category of pig (Friedman χ^2^ = 8.22, p < 0.05, Fig. 5e) and tended to be higher in the victims compared to the controls (p = 0.08). Family_XIII_AD3011_group (Friedman χ^2^ = 7.6, p < 0.05, Fig. 5f) was higher in the biters than in the controls (p < 0.05). *Ruminiclostridium*_9 (Friedman χ^2^ = 6.89, p < 0.05, Fig. 5g) tended to be higher in the biters than in the control (p = 0.08).

### Faecal short chain fatty acids

Faecal acetate concentrations were associated with category of pig (F_(2,5.4)_ = 6.8, p < 0.05, Fig. 6a), with the biters having significantly lower concentrations than the victim pigs (p < 0.05). Acetate was also significantly higher on the commercial farm (65.8 ± 4.6 mmol/L) than on the experimental farm (41.8 ± 1.8 mmol/L, F_(1,10.6)_ = 17.9, p < 0.01). Faecal propionate concentrations were associated with category of pig (F_(2,12.1)_ = 6.4.0, p < 0.05, Fig. 6b), with lower propionate concentrations in the biters than the victims (p < 0.05) and to a lesser extent the control pigs (p < 0.1). Butyrate concentrations were associated with category of pig (F_(2,5.6)_ = 12.2, p < 0.01, Fig. 6c), with lower concentrations in both biters (p < 0.05) and victims (p < 0.04) than controls. Iso-butyrate concentrations were associated with category of pig (F_(2,19.2)_ = 4.0, p < 0.05, Fig. 6d), with biters tending to have lower concentrations than the controls (p < 0.1). There was also a main effect of sex, with females (1.0 ± 0.15 mmol/L) having higher iso-butyrate concentrations than males (0.5 ± 0.11 mmol/L, F_(1,23.6)_ = 9.3, p < 0.01). Furthermore, iso-butyrate concentrations were higher on the commercial farm (1.0 ± 0.3 mmol/L) than on the experimental farm (0.5 ± 0.1, F_(1,12.0)_ = 6.5, p < 0.05). Valerate concentrations were associated with category of pig (F_(2,9.7)_ = 4.6, p < 0.05, Fig. 6e), with lower concentrations in the biters (p < 0.05) than in the controls.

The molar proportion of acetate was associated with tail biting (F_(2,18.3)_ = 11.0, p < 0.001, Fig. 7a), with the biters having (p < 0.05) and victims (p = 0.09) tending to have a higher proportion than the controls. The molar proportions of acetate were higher on the commercial farm (0.59 ± 0.01) than on the experimental farm (0.53 ± 0.01, F_(1,7.4)_ = 13.5, p < 0.01). The molar proportions of butyrate were also associated with category of pig (F_(2,6.7)_ = 9.4, p < 0.01, Fig. 7b), with the proportion being higher in the control group than in the biters (p < 0.05) and tending to be higher than the victims (p < 0.1). The molar proportions of iso-butyrate tended to be affected by tail biting category (F_(2,20.6)_ = 2.7, p = 0.05, Supplement Fig. 1a) and were higher in the females (0.011 ± 0.001) than the males (0.005 ± 0.009, F_(1,22.7)_ = 9.0, p < 0.01). The molar proportion of valerate was also associated with category (F_(2,18.6)_ = 3.8, p < 0.05, Supplement Fig. 1b), although we could not detect any differences between biter, victim or control pigs in the posthoc test.

### Plasma short-chain fatty acids

Butyrate was associated with category of pig (F_(2,11.6)_ = 4.7, p < 0.05, Fig. 8a), with the biters having higher concentrations than the victims (p < 0.05). Iso-butyrate concentrations were also associated with category (F_(2,15.8)_ = 4.2, p < 0.05, Fig. 8b), with the control pigs having higher concentrations than the victims (p < 0.05). Valerate concentrations were also associated with category (F_(2,10.5)_ = 5.5, p < 0.05, Fig. 8c), with the biters having higher concentrations than the victims (p < 0.05). Iso-valerate concentrations were associated with category (F_(2,13.8)_ = 3.9, p < 0.05, Fig. 8d), with the controls tending to have higher concentrations than the victims (p = 0.051). The molar proportions of plasma SCFA were not associated with category of pig nor farm (data not shown).

## Discussion

The aim of this study was to determine whether matched sets of pigs that were tail biters, victims of tail biting, or control pigs that were not involved in tail biting had a different microbiota composition, diversity and SCFA profile. Our results showed that the gut microbiota of tail biter pigs was more diverse than the control pigs. Furthermore, the gut microbiota composition within each tail biting set was most different between the biter and the control pigs, with a higher relative abundance of Firmicutes in tail biter pigs than the controls. This study also provides the first evidence of an association between tail biting and faecal and plasma SCFA.

The higher relative abundance of Firmicutes in the tail biter pigs was primarily driven by the higher abundance of the families Lachnospiraceae, Ruminococcaceae and Clostridiales Family XIII. Our results are partly in line with the so far only published paper on gut microbiota composition and tail biting in pigs^27^. In line with their results, we also found the highest similarity in microbiota composition between the biters and the victims and the largest differences between biters and controls. By contrast, we found a higher microbial alpha diversity in the tail biting pigs, whereas the study by Rahbi et al. did not. A high microbial diversity is commonly interpreted as favourable as it enables the community to have a greater ability to respond to disturbances^56^. However, all pigs in our study had reasonably high alpha diversity values, and therefore, the higher diversity in the biters may not necessarily be beneficial^57^.

In agreement with the study by Rabhi et al.^27^, we identified taxa differences between biter pigs and controls. Rabhi et al., reported lower *Lactobacillus* in the biter pigs, and a similar reduction in the abundance of *Lactobacillus* has also been reported in feather pecking hens^58^. A lower abundance of *Lactobacillus* has been linked to high psychological stress in a number of species^18,59,60^. However, we did not find any effect of tail biting on *Lactobacillus* in our study. We did find a higher relative abundance of several families within the Clostridiales order in the biter pigs, which is in line with previous studies in laying hens involved in feather pecking^58,61^. Clostridiales regulate microbial gut-derived T-cell immune and serotonergic signals through which they can affect behavior^62–64^. Increases in the relative abundance of the Clostridiales order has also been linked to a number of behavioural disorders such as autism^62^, schizophrenia^65^ and as a consequence of social stress^18,66^. At family level, we found increased levels of Lachnospiraceae, Ruminococcaceae and Clostridiales Family XIII in the tail biter pigs. Members of the Clostridiales order and in particular within the Ruminococcaceae and Lachospiraceae families, have been associated with serotonin biosynthesis via production of 5-HT^67^. An increase in serotonin metabolism (increased 5-HIAA levels) has also been detected in the prefrontal cortex of biter pigs^12^. Ruminococcaceae and Lachnospiraceae families are also important for the production of SCFA and in particular butyrate^68^.

This study also provides the first evidence of an association between tail biting and faecal and plasma SCFA. Faecal SCFA concentrations were lowest in the biting pigs. In addition, there was a shift towards increased faecal molar proportions of acetate but reduced proportions of butyrate in the biter pigs, and to a lesser extend also in the victim pigs. The lower faecal concentrations of SCFA in biter pigs (and to a lesser extent victim pigs) could indicate an increased colonic absorption^69^, or an altered rate of passage of the digesta^28,70^. However, the lower concentrations of SCFA in the biter pigs seems contradictory to the higher abundance of SCFA producing microbial families. Other studies have also found altered SCFA in humans and animals with behavioural disorders. For example, lower faecal acetate^32,71^, propionate^32,71^ and butyrate^71^ concentrations have been observed in autistic children compared to healthy controls, although increased faecal SCFA in autism has also been reported^72^. Mice subjected to social stress had lower colonic concentrations of acetate and butyrate but increased concentrations of propionate^73^. However, hens that feather pecked had a higher concentrations of total caecal SCFA and a reduced proportion of acetate compared to low feather pecking birds^74^.

Most butyrate is used as an energy source by the colonic mucosa^75^, and acetate and propionate are metabolized by the liver as a substrate for lipogenesis and gluconeogenesis^76,77^. Therefore, only a minor fraction of colonic produced SCFA reaches the circulation, and plasma concentrations of SCFA are not necessarily a good reflection of faecal concentrations^78^. We found increased plasma concentrations of butyrate and valerate in biter pigs compared to the victims and increased iso-butyrate and iso-valerate in the control pigs compared to victim pigs, although we could not detect any major shifts in the molar proportion of plasma SCFA related to tail biting. The differences in plasma SCFA profiles between biter and victim pigs suggest that they may be physiologically different from each other, even though they are kept in the same pens, and it would be valuable to further look into how plasma SCFA profiles play a role in the development of tail biting.

The SCFA profiles were not consistent in relation to tail biting in blood and faeces, with lower faecal concentrations of SCFA in the biters, but lower plasma SCFA in victim pigs. It has been suggested that circulating concentrations of SCFA may be better indicators of metabolic health than faecal SCFA^78,79^, and therefore, the lower plasma concentrations of SCFA may suggest poorer metabolic health in the victim pigs. The circulating SCFA may influence the brain locally, but on the other hand, it has also been shown that colonic SCFA can impact the brain remotely via interaction with receptors GPR 43/41 in the colonic mucosa that release Glucagon-like Peptide 1 and Peptide YY which influence the signalling to the brain via the vagus nerve^80^.

The different SCFA can have varying impacts on behaviour. Butyrate administration has been shown to have anti-depressant effects in mice^81–83^. When SCFA in the colon were experimentally increased by feeding acylated starches in mice, it was shown that higher acetate concentrations, but not butyrate or propionate, led to a reduction in anxiety-like behaviours, suggesting that acetate may have a beneficial impact on host behaviour^84^. Chronically stressed mice orally supplemented with a mixture of acetate, propionate and butyrate showed normalized reward-seeking behaviour and increased stress responsiveness^85^. On the other hand, propionate administration directly into the brain impairs social behavior^86,87^ and peripheral administration increases anxiety and repetitive behaviours^88^. These studies suggest that faecal acetate and butyrate may be associated with reduced depression- and anxiety-like behaviours, but that propionate may be associated with abnormal social behaviours. However, the biter pigs in our study had lower faecal concentrations of acetate, butyrate and propionate. Studies assessing the effects of SCFA on behaviours vary widely in SCFA measurement location (i.e., colonic, cecal, faecal, circulating) as do studies administering SCFA (i.e., orally, intra-gastric, intra-colonic, intraperitoneal) and this is likely to affect the outcomes. It is also worth noting that the SCFA production in the colon and their absorption by the colonic epithelium is a dynamic process, and the measurements in stool or blood samples will display a snapshot of how the situation looks at a given time point. Despite this, our study is the first to suggest reduced faecal SCFA in biter pigs, and reduced circulating SCFA in victim pigs. Further research would be necessary to confirm any cause and effect relationships between circulating and faecal SCFA and tail biting behaviour.

To ensure that the control pig was not involved in tail biting, we chose a control pig from a different but nearby pen with the same diet and management. Thus, we cannot rule out that subtle differences between the pen environments or the stress of being housed in a pen with an ongoing episode of tail biting contributed to the observed differences. Tail biting and victim pigs, on the other hand, come from the same pen and so the differences between these two categories could be especially relevant. However, even here there are potential difficulties in interpretation, since in three sets, the biter pigs were also victims themselves. Neither can we rule out that more victim pigs would have been classified as biter pigs if we had observed them more often or had left the biters in the pen for a longer period of time. We removed the biter pigs from the pens at the first signs of tail damage for ethical reasons. We used the same criteria to identify biter pigs as in previous studies, e.g., when a pig was clearly observed biting another pig with visible tail damage at least five times (e.g.^37^). However, many tail biting relationships are mutual, with two pigs biting each other^38^. In some studies, as many as 98% of the pigs in a pen are involved in tail biting^37^, although other studies have found that some pigs will not engage in tail biting (e.g., a tail biting resistant phenotype^89^). In future studies, it may be valuable to create four categories of pigs instead (true’ biter without tail damage, biter/victim, ‘true’ victim never observed biting and ‘true’ control), but more pigs would be required for such a study. Finally, in one set it was difficult to identify the biter, and we suggest more extensive monitoring by video cameras as long as this can be done in an ethical way so that further damage can be prevented.

We collected data on two different farms, because the occurrence of tail biting was relatively low and it was difficult to get enough episodes. We speculate that the reason for the low occurrence was that several factors known to protect against the development of tail biting (e.g., low stocking densities^9^, environmental enrichment^8^ good environmental conditions^7,9^) were present on the farms from which we recruited our pigs.

While we observed differences between biter pigs and controls in microbiota composition within the matched sets, the farm environment and/or diet had a larger impact on the microbiota composition than tail biting category. We found some differences in SCFA profiles between the farms, and the differences between tail biting categories appeared to be larger—but in the same direction—on the commercial farm than on the experimental farm. The diet, such as the phosphorus content^90^ and the amount of fibre^91–93^ has an impact on SCFA and microbiota, and differences in the diet most likely explained the differences between farms. Despite the diet and other differences in environment and management, the results on both farms were in agreement with each other, which strengthens the findings of this study.

The pigs in our study were housed in mixed-sex pens with females and castrated males. The majority of the victim pigs were males, which is in agreement with previous studies^5^. However, previous studies have reported that females are more likely to be biters^94^. Females become more active when reaching sexual maturity and may re-direct their behaviour towards biting inactive male castrates because of lack of stimulation^5^. However, we observed that most biter pigs were also males. We also found some sex differences with higher levels of faecal (but not plasma) concentrations iso-butyrate in females, independent of tail biting category. The reasons for this is not clear, but could be due to the small number of females in the study, and would need further investigation.

In conclusion, we identified differences in microbiota composition and diversity as well as in faecal and plasma SCFA profiles between biter, victim and control pigs. Within each matched set, the gut microbiota composition was most different between the biter and the control pigs, with a higher relative abundance of Firmicutes in tail biter pigs and this difference was primarily driven by the higher abundance of the families Lachnospiraceae, Ruminococcaceae and Clostridiales Family XIII. However, other factors, such as the farm and diet, had a larger impact on the gut microbiota composition than the category of the pig. We also found reduced concentrations of SCFA in the faeces of biter pigs as well as reduced plasma SCFA concentrations in the victim pigs. This study provides further evidence for the role of the gut microbiota composition and diversity, and now also for the role of SCFA, in tail biting in pigs. Future studies need to confirm these associations and determine the potential causality.

## Supplementary Information

Supplementary Information.

Supplementary Figure 1.

## Data Availability

The datasets generated and analysed for the current study are available in the Open Science Framework repository (https://osf.io/j5g9q/).
