# Supplementary material for: The gut microbiota and microbial metabolites are associated with tail biting in pigs
Source: Sci Rep. 2021 Oct 15;11:20547. doi: 10.1038/s41598-021-99741-8 (PMC8521594; doi:10.1038/s41598-021-99741-8)
Supplement: Supplementary file 1 — Supplementary Information. [file 41598_2021_99741_MOESM1_ESM.docx]

**Supplement 1. Analysis of plasma SCFA:**

Authentic standards for SCFAs were obtained, Formic Acid (C1) (Scharlau), acetic acid (C2) (Honeywell), propionic acid (C3) (Alfa Aesar), butyric acid (C4) (Sigma Aldrich), isobutyric acid (C4) (Alfa Aesar), Succinic acid (C4) (Acros), isovaleric acid (C5) (Sigma Aldrich), valeric acid (C5) (Alfa Aesar) and caproic acid (C6) (Sigma Aldrich). Analytical reagent-grade 3-nitrophenylhydrazine (3NPH)-HCl (97%), 2-nitrophenylhydrazine N-(3-dimethylaminopropyl)-N0-ethylcarbodiimide (EDC) HCl, quinic acid, HPLC grade pyridine and Lichrosol reagent grade MeOH and water was obtained from Sigma–Aldrich. Acetonitrile Optima LCMS Grade was obtained from Fisher scientific. ^13^C6-3NPH-HCl was custom synthesized to us by IsoSciences Inc. (King of Prussia, PA, USA) (catalouge 13309). According to the accompanying certificate of analysis, this custom-synthesized compound was structurally confirmed by 1H NMR spectroscopy and by MS/MS on a triple-quadruple mass spectrometer.

**Analysis of SCFA in plasma.**

SCFA in plasma were analyzed according to ^1^. Briefly, fifty microliters of a mixed standard solution containing 4 mM of formic acid and acetic acid, 2 mM of propionic acid, and 1 mM of each of the other six SCFAs were added to a 2 mL borosilicate test tube that contained 1 mg of ^13^C6-3NPH HCl. Twenty-five microliters of 120 mM EDC-6% pyridine solution and twenty-five microliters 75% MeOH were then added to the mixture. The mixture was reacted at 4°C for 4 hours. Twenty-five microliters quinic acid in MeOH was added and quenching proceeded for 45 min. After quenching, the mixture was transferred to a volumetric flask with 10% MeOH and diluted with the same solvent to 100 mL. This solution was used as the internal standard mix and was stored in aliquots at -20°C. SCFAs were analyzed in plasma by LC-MS according to a method described before ^2^ with minor modifications. In total, 10 μl plasma was incubated with 60 μl 75% methanol, 10 μl 200 mM 3-NPH and 10 μl 120 mM EDC-6% pyridine at ambient temperature for 45 min with shaking. The reaction was quenched by addition of 10 μl of 200 mM quinic acid (15 min with shaking). The samples were centrifuged at 15 000 g for 5 min and the supernatant moved to a new tube. The samples were made up to 1 mL by 10% methanol in water and again centrifuged at 15 000 g for 5 min. In total, 100 μl of the derivativesed (^12^C 3NPH) sample was mixed with 100 μl of labelled (^13^C 3NPH) internal standard. A mixed external standard solution containing 3,2 µM – 0,63 nM of formic acid and acetic acid, 3,2 µM – 0,31 nM of propionic acid, and 0,8 µM – 0,16 nM of each of the other six SCFAs were always prepared fresh and used for each batch. Samples were analyzed by a 6500+ QTRAP triple-quadrupole mass spectrometer (AB Sciex, 11432 Stockholm, Sweden) which was equipped with an APCI source and operated in the negative-ion mode. Chromatographic separations were performed on a Phenomenex Kinetix Core-Shell C18 (2.1, 100 mm, 1.7 um 100Å) UPLC column with SecurityGuard ULTRA Cartridges (C18 2.1mm ID) (changed at regular intervals at). The column was backflushed for 60 min between each batch to ensure good chromatographic separation. Water (100% solvent A) and acetonitrile (100% solvent B) was the mobile phases for gradient elution. The column flow rate was 0.4 mL/min and the column temperature was 40°C, the autosampler was kept at 4°C. LC starting conditions at 0.5% B, held for 3 min, 3 min 2.5% B ramping linearly to 17% B at 6 min, then to 45% B at 10 min and 55% B at 13 min. Followed by a flush (100% B) and recondition (0.5% B), total runtime 15 min. The MRM transitions were optimized for the analytes one by one by direct infusion of the derivatives containing 10 µM of each fatty acid, essentially as according to ^2^. The Q1/Q3 pairs were used in the MRM scan mode to optimize the collision energies for each analyte, and the two most sensitive pairs per analyte were used for the subsequent analyses. The retention time window for the scheduled MRM was 1 min for each analyte. The two MRM transitions per analyte, the Q1/Q3 pair that showed the higher sensitivity was selected as the MRM transition for quantitation. The other transition acted as a qualifier for the purpose of verification of the identity of the molecule. UPLC/MRM-MS data was acquired in the “scheduled MRM” mode using the Analyst 1.5 software and data processing was performed using the MultiQuant 3.0.3 software (AB Sciex, 11432 Stockholm, Sweden).

References:

1 Fristedt, R., Rupert, V., Trower, T., Cooney, J. & Landberg, R. Hyphenated quantitation of circulating short-chain fatty acids in blood from different organisms and their biological variation. *In preparation* (2021).

2 Han, J., Lin, K., Sequeira, C. & Borchers, C. H. An isotope-labeled chemical derivatization method for the quantitation of short-chain fatty acids in human feces by liquid chromatography–tandem mass spectrometry. *Analytica chimica acta* **854**, 86-94 (2015).
