# Supplementary material for: The gut microbiota and microbial metabolites are associated with tail biting in pigs
Source: Sci Rep. 2021 Oct 15;11:20547. doi: 10.1038/s41598-021-99741-8 (PMC8521594; doi:10.1038/s41598-021-99741-8)
Supplement: Supplementary file 2 — Supplementary Figure 1. [file 41598_2021_99741_MOESM2_ESM.docx]

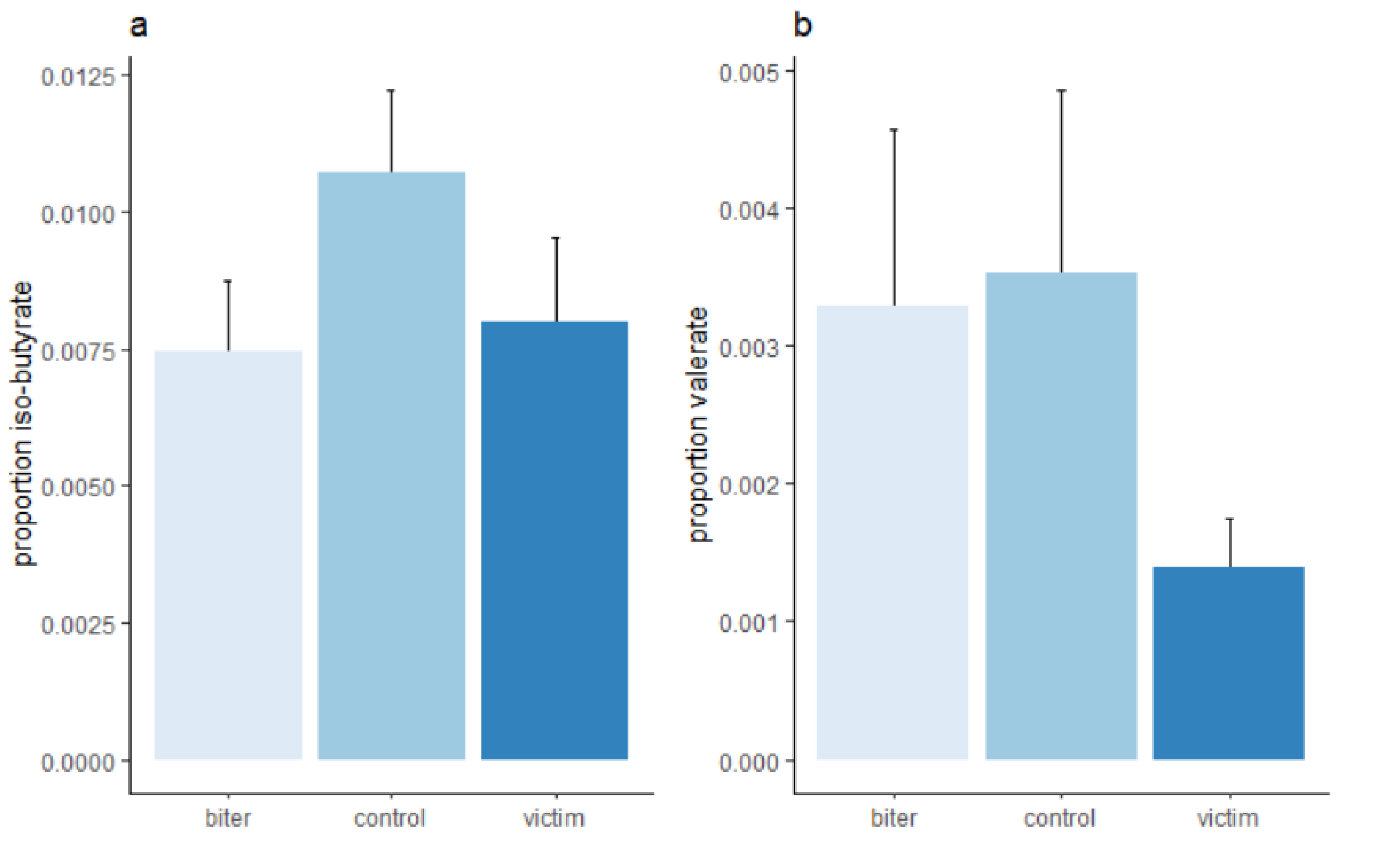


**Supplement figure 1.** Molar proportions of faecal SCFA (mean ± sem) in the biters, victims and control pigs. Panel **a.** shows the molar proportion of iso-butyrate and panel **b.** shows the molar proportion of valerate.
